# Chemoradiotherapy with Brachytherapy or Electron Therapy Boost for Locally Advanced Squamous Cell Carcinoma of the Anus—Reducing the Colostomy Rate

**DOI:** 10.1007/s12029-016-9850-4

**Published:** 2016-07-14

**Authors:** C. Kent, E. M. Bessell, J. H. Scholefield, S. Chappell, L. Marsh, J. Mills, I. Sayers

**Affiliations:** 1 Department of Clinical Oncology, Nottingham, UK; 2 Department of Surgery, Nottingham, UK; 3 School of Life Sciences, University of Nottingham, Nottingham, UK

**Keywords:** Anal cancer, Stoma formation, Boost, Brachytherapy, Electron beam therapy, Radiotherapy

## Abstract

**Purpose:**

The aim of this study is to determine overall survival, disease-specific survival and stoma-free survival after treatment of squamous cell carcinoma of the anus with chemoradiotherapy followed by brachytherapy or electron boost in a recent cohort of patients.

**Methods:**

Fifty-two patients (median age 62 years) were treated with radical chemoradiotherapy (mitomycin C, infusional 5-fluorouracil concurrently with conformal radical radiotherapy 45 Gy in 25 fractions over 5 weeks) followed by a radiotherapy boost between 1 December 2000 and 30 April 2011. Follow-up was to 30 November 2014. Thirty-six patients received a boost (15–20 Gy) over 2 days with ^192^Ir needle brachytherapy for anal canal tumours, and 16 patients received electron beam therapy (20 Gy in 10 fractions in 2 weeks) for anal margin tumours. A defunctioning stoma was only created prior to chemoradiotherapy for fistula or severe anal pain.

**Results:**

The overall survival for the 36 patients treated with chemoradiotherapy followed by brachytherapy was 75 % (95 % CI, 61–89) at 5 years, the disease-specific survival was 91 % (95 % CI, 81–101 %), and the stoma-free survival was 97 % (95 % CI, 91–103 %) all at 5 years. For the 16 patients treated with an electron boost for anal margin tumours, the 5-year overall survival, disease-specific survival and stoma-free survival were 68 % (95 % CI, 44–92 %), 78 % (95 % CI, 56–100 %) and 80 % (95 % CI, 60–100 %), respectively.

**Conclusions:**

A very low stoma formation rate can be obtained with radical chemoradiotherapy followed by a brachytherapy boost for squamous cell carcinoma of the anal canal but not with an electron boost for anal margin tumours.

## Introduction

Squamous cell carcinoma (SCC) of the anus is caused predominantly (84 %) by human papilloma virus (HPV 16) in comparison to cervical carcinoma (70 %) (HPV 16 and 18) and oropharyngeal carcinoma (33 %) (HPV 16) [1]. In contrast to the high doses of radical radiotherapy (65–70 Gy) used in the chemoradiotherapy of cervical carcinoma and oropharyngeal carcinoma, recent trials particularly in the UK ACT II trial [2] have established that doses of 50.4 Gy in 28 daily fractions combined with mitomycin C and 5-fluorouracil give a 3-year progression-free survival of 80 % for T1 or T2 disease but 65 % for T3 or T4 disease. Similarly, the 3-year colostomy-free survival was 84 % for T1 or T2 disease and 61 % for patients with T3 or T4 disease. In the RTOG 98-11 trial [3] published in 2008, in which a radiotherapy boost of 10–14 Gy in 2 Gy fractions was given for locally advanced disease (total 55–59 Gy), the 5-year colostomy-free survival was 90 % in the Mitomycin-based group. European guidelines for diagnosis, treatment, and follow-up of anal cancer have recently been published [4]. The results of chemoradiotherapy with brachytherapy or electron therapy boost in Nottingham, UK (2000–2011), for locally advanced SCC of the anus were analysed to determine overall survival, disease-specific survival and stoma-free survival. The patients were treated during the modern era with discussion of patients at a multidisciplinary team meeting and treatment by a specialist surgeon and oncologist.

## Methods

All patients referred to Nottingham City Hospital, UK, between 1 December 2000 and 30 April 2011 for chemo/radiotherapy for SCC of the anal canal or margin were identified. No patient was documented with HIV infection. These patients had all been discussed at the Colorectal Cancer Multi-disciplinary Team Meetings (which started in the year 2000) held at Queens Medical Centre, Nottingham, which included patients with anal cancer. These patients were recorded on an electronic database. All patients gave informed written consent to treatment. The project was considered by the Nottingham Research Ethics Committee as an audit and hence was ethically approved as part of a cohort of audits undertaken at the time by Nottingham University Hospital NHS Trust. An individual ethical assessment of this project was not deemed necessary. In addition, ethical approval was obtained from Trent Cancer Registry to access data on patients with anal cancer. The lead surgeon, J. Scholefield, and the lead oncologist, E. Bessell, were responsible for treating the vast majority of these patients. All the patients were treated using standard departmental protocols (no extra treatments or investigations were carried out).

The staging investigations were as follows: the local extent of the anal cancer was determined by digital examination and proctoscopy. The position of the tumour in the lithotomy position was recorded with 12 o’clock representing the anterior position and 6 o’clock representing the posterior position. The distance from the anal verge was measured and also the length of the tumour. Magnetic resonance imaging and endorectal ultrasound examination were not used. All patients had a CT scan of chest, abdomen and pelvis, and a fine needle aspirate was obtained from suspicious inguinal lymph nodes for cytological examination.

No defunctioning stoma was formed unless deemed essential. Patients were advised to have a low-roughage diet during treatment.

For each patient, information was obtained on age, sex, TNM stage, histology, the date of diagnosis and the date of starting treatment, the chemotherapy used, the radiotherapy dose both external beam and boost, the use of defunctioning colostomy and the date of any salvage surgery. The patients were followed 3-monthly for 2 years and 4–6-monthly until a minimum of 5 years of follow-up. Local recurrence was assessed initially by digital examination and confirmed histologically and by magnetic resonance imaging (MRI). Patients with confirmed local recurrence were investigated further with a CT scan of chest, abdomen and pelvis.

Anal function was assessed by Common Terminology Criteria for Adverse Events (CTCAE) version 4.0.

Early-stage disease was considered to be T1 N0 or T2 (<3 cm in diameter) N0. All other stages were considered to be locally advanced.

### Treatment

Patients with early-stage disease were considered for entry into the ACT II trial [2] which recruited between June 2001 and December 2008. Following the closure of this trial, standard treatment for early-stage disease was mitomycin/fluorouracil concurrently with external beam radiotherapy to a total dose of 50.4 Gy in 28 daily fractions over 5.5 weeks.

Patients with locally advanced disease were treated with mitomycin/fluorouracil according to the protocol of the ACT I trial [5]. The radiotherapy was also prescribed according to this protocol. The standard pelvic field was defined with superior border 2 cm above the inferior aspect of the sacroiliac joints, the inferior border 3 cm below the anal margin or 3 cm below the inferior extent of the anal tumour. The lateral aspects were arranged to cover the inguinal nodes with no routine shielding of the femoral heads. The mid line dose was 45 Gy in 25 fractions (1.8 Gy per fraction) in 5 weeks with an anterior-posterior field arrangement.

A radiotherapy boost of 15–20 Gy over 2 days was given with low dose rate ^[192]^Ir brachytherapy for anal canal tumours. Five needles were used covering the hemicircumference containing the tumour, sparing the other hemicircumference in order to preserve sphincter function. A specially designed template was used in the majority of patients. A radiotherapy boost of 20 Gy in ten fractions in 2 weeks was given with high energy electrons to anal margin tumours.

### Statistical Analysis

Overall survival, disease-specific survival and stoma-free survival were estimated using the Kaplan-Meier method with IBM SPSS Statistics version 21.

## Results

A total of 92 patients were found on the database for the period 1 December 2000 to 30 April 2011 (see Table 1). Follow-up was to 30 November 2014. At that date, 50 of the 92 patients were alive without recurrence (median follow-up 7.2 years, range 3–14 years). There was female predominance as expected with 60 females and 32 males. The seven patients treated with palliative intent were so treated because of advanced age, medical comorbidities or extensive local or metastatic disease. There were also eight patients not fit enough for chemotherapy who received radical external beam radiotherapy (three of these had an iridium boost). Twenty-five patients received chemoradiotherapy with no boost. This was a mixed group of patients including those entered into the ACT II trial or treated by the ACT II radiotherapy protocol, those refusing a boost often because of a prolonged acute radiation reaction and those too frail for further treatment. There were therefore 52 patients receiving radical chemoradiotherapy with a boost (36 iridium: 16 electron).

**Table 1:** Demographic characteristics of the patients studied

Whole group, *n* = 92				
Age (median) 59.5 years				
Gender:	Male	32 (35 %)		
	Female	60 (65 %)		
Treatment intent:	Palliative	7	Radical	85
N-stage	T-stage	No. of patients	% of total	
N0	T1	19	20.7	
N0	T2	37	40.2	
N0	T3	20	21.7	
N0	T4	6	6.5	
N1	T3	1	1.1	
N1	T4	4	4.4	
N2	T3	3	3.3	
N3	T3	1	1.1	
N3	T4	1	1.1	
Radical treatment				
Chemoradiotherapy plus boost, *n* = 52				
Anal canal		Anal margin		
Iridium boost, *n* = 36		Electron boost, *n* = 16		
Age (median) 61 years		Age (median) 63 years		
Stage	No. of patients (%)	Stage	No. of patients (%)	
T1 N0	3 (8.3)	T1 N0	4 (25)	
T2 N0	17 (47.2)	T2 N0	7 (43.8)	
T3 N0	14 (38.9)	T3 N0	2 (12.5)	
T2 N2	1 (2.8)	–	–	
T3 N2	1 (2.8)	–	–	
–	–	T4 N0	2 (12.5)	
–	–	T4 N1	1 (6.2)	
Radical treatment (other)				
External beam radiotherapy alone	8			
Chemoradiotherapy without boost	25			

### Radical Chemoradiotherapy with Iridium Brachytherapy Boost

**Fig. 1:**
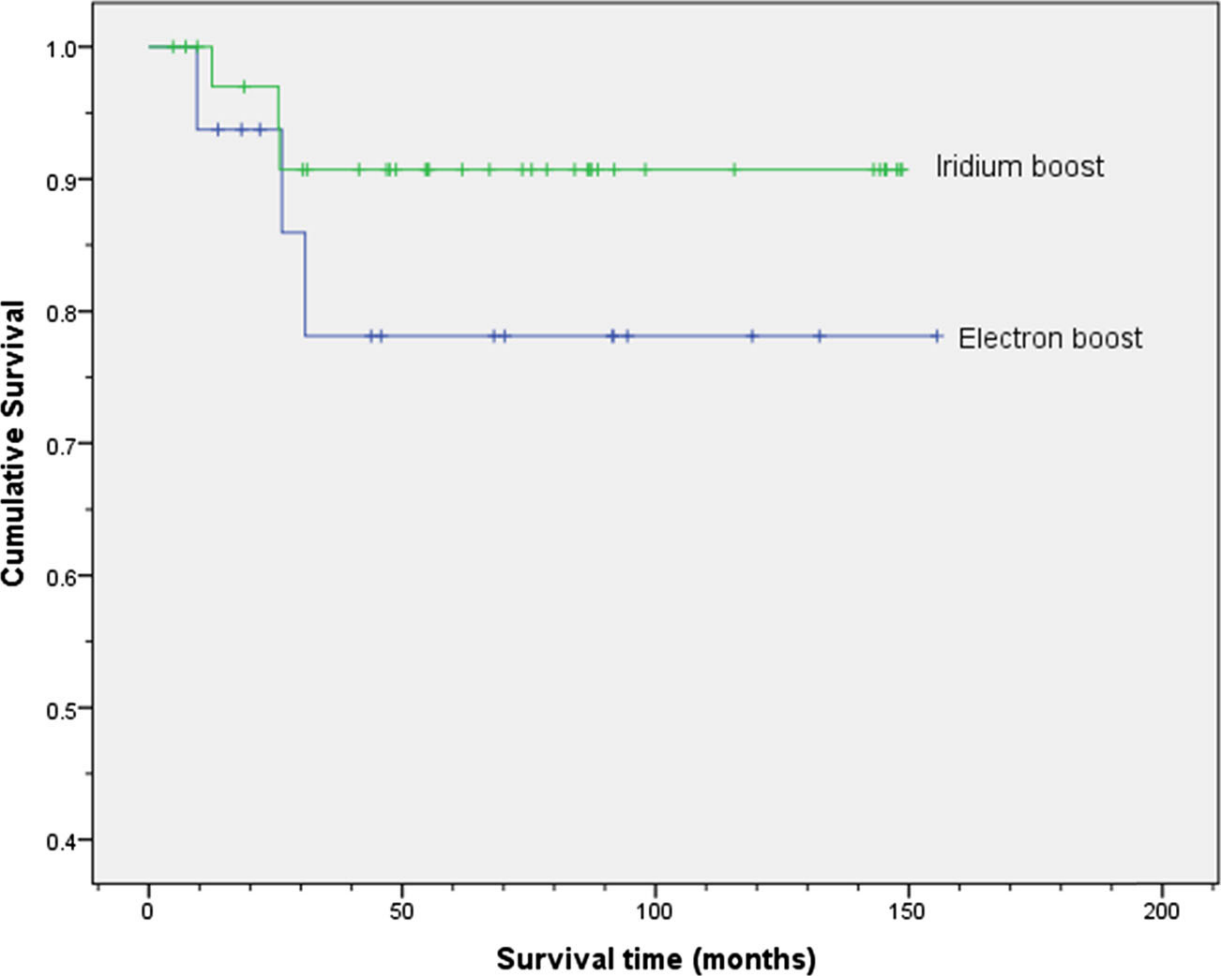
Kaplan-Meier plot showing disease-specific survival for the groups receiving radical chemoradiotherapy with either the iridium boost or electron boost. The cumulative proportion surviving at 5 years (95 % CI) was 0.91 (0.81, 1.01) for the iridium group and 0.78 (0.56, 1.00) for the group receiving the electron boost. The log-rank test showed no statistically significant difference between the two groups (*p* = 0.272)

**Fig. 2:**
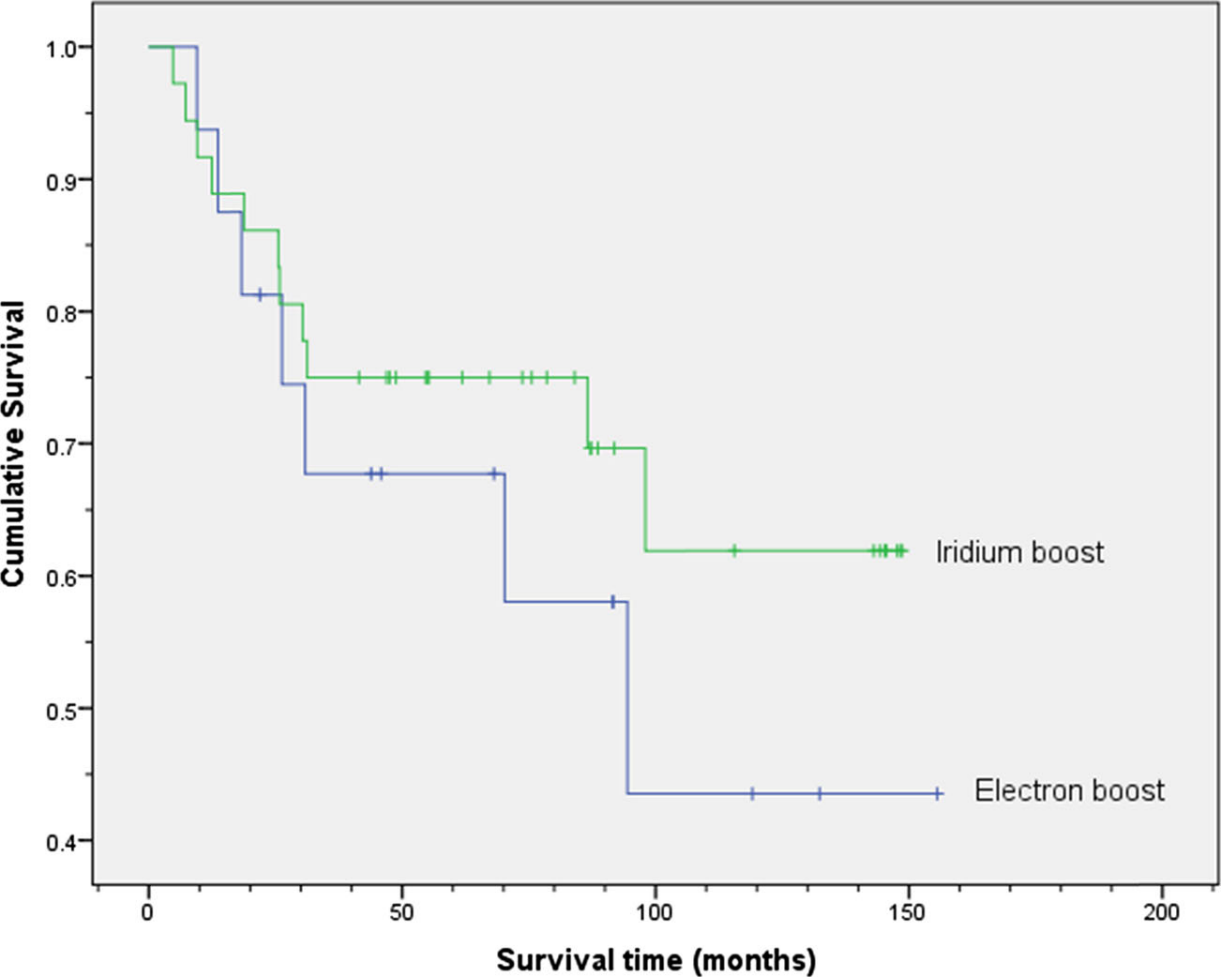
Kaplan-Meier plot showing overall survival for the groups receiving radical chemoradiotherapy with either the iridium boost or electron boost. The cumulative proportion surviving at 5 years (95 % CI) was 0.75 (0.61, 0.89) for the iridium group and 0.68 (0.44, 0.92) for the group receiving the electron boost. The log-rank test showed no statistically significant difference between the two groups (*p* = 0.375)

The disease-specific survival for these 36 patients (Fig. 1) was 91 % (95 % CI 81–101) at 5 years with only one local recurrence and two additional patients developing metastatic disease. One patient had a defunctioning stoma which was formed prior to treatment and not reversed. The overall survival was 75 % (95 % CI 61–89) at 5 years (Fig. 2). The patient with local recurrence only had a salvage abdomino-perineal resection with a stoma but died of disease 15 months later.

### Radical Chemoradiotherapy with Electron Boost

The disease-specific survival for these 16 patients (Fig. 1) was 78 % (95 % CI 56–100) at 5 years with two local recurrences and one additional patient who developed metastatic disease. Only one patient had a stoma formed before chemoradiotherapy for a T4 tumour, and this was not reversed.

The overall survival was 68 % (95 % CI 44–92) at 5 years (Fig. 2). The two patients who developed local recurrence had salvage abdomino-perineal resection with a stoma but both died of metastatic disease.

It was not the intention of this retrospective study to compare the survival of patients with anal canal tumours with those with anal margin tumours, but the log-rank test showed no statistically significant difference between the electron boost group and the brachytherapy group as far as disease-specific survival (*p* = 0.272) and overall survival (*p* = 0.375) is concerned.

### Toxicity

The toxicity from chemoradiotherapy for anal cancer is well documented from the large randomised controlled trials. Only 2 of the 52 patients receiving radical chemoradiotherapy with a boost developed prolonged grade 3 toxicity, and one of these was later found to be associated with local recurrence.

### Anal Function and Stoma Formation

**Fig. 3:**
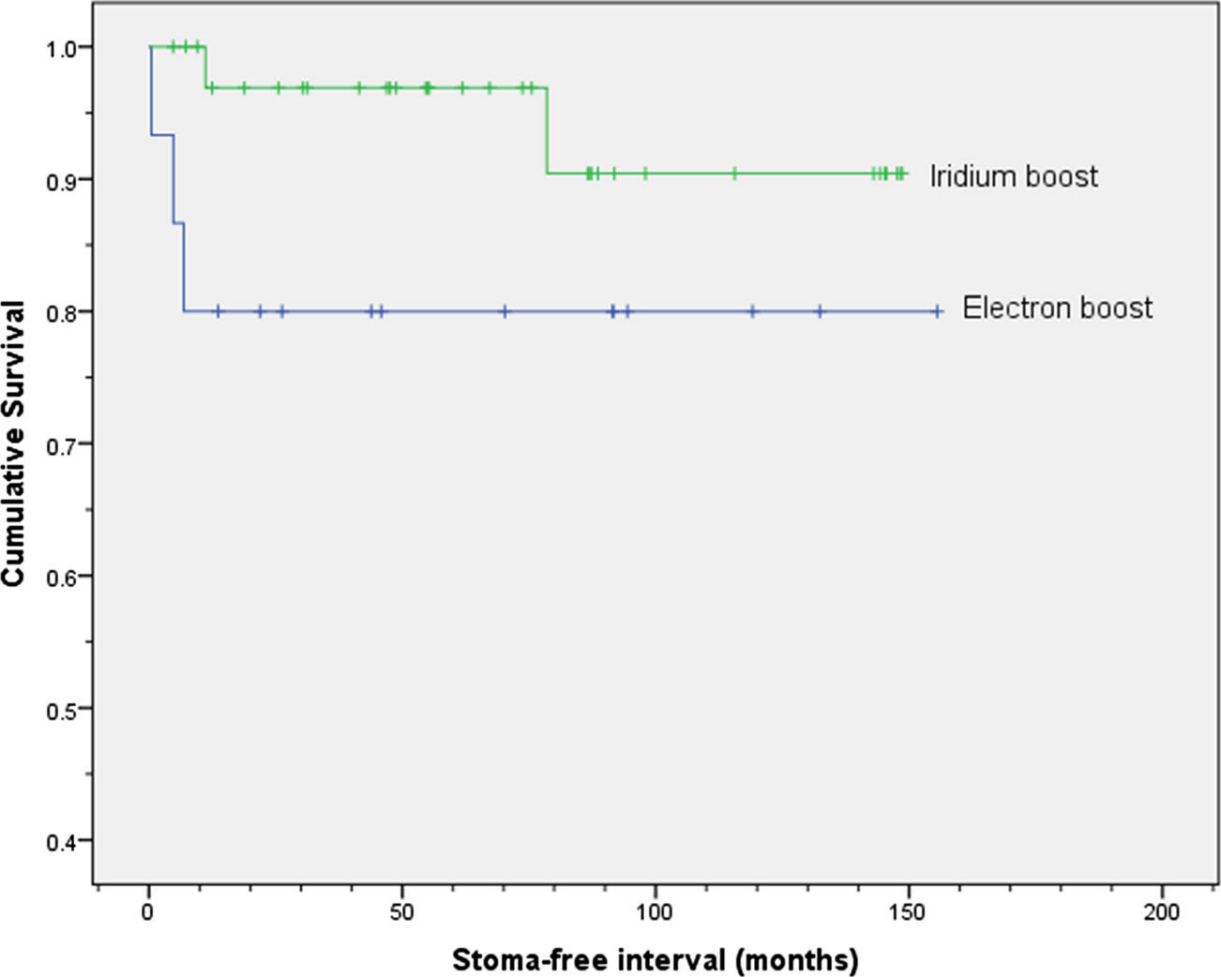
Kaplan-Meier plot showing stoma-free survival for the groups receiving radical chemoradiotherapy with either the iridium boost or electron boost. The cumulative proportion surviving stoma-free at 5 years (95 % CI) was 0.97 (0.91, 1.03) for the iridium group and 0.80 (0.60, 1.00) for the group receiving the electron boost. The log-rank test showed no statistically significant difference between the two groups (*p* = 0.119)

Two of the 52 patients treated with radical chemoradiotherapy and a boost had a stoma formed before treatment which was not reversed, and 3 patients had a stoma formed because of abdomino-perineal resection for local recurrence but died of disease. There were therefore 47 patients without a stoma. The stoma-free survival at 5 years was 97 % (95 % CI 91–103) (Fig. 3) for the 36 patients who received brachytherapy as a boost and 80 % (95 %, CI 60–100) for the 16 patients who received electron therapy as a boost. These stomas were all cancer rather than treatment-related, and therefore, the cancer-related stoma rates were 3 % at 5 years for the brachytherapy group and 20 % at 5 years for the electron group. The reasons for the defunctioning colostomy in the two patients who had a stoma formed prior to definitive chemoradiotherapy was in one, severe anal pain requiring inpatient care with poor control with analgesics and in the other a rectovaginal fistula.

There were five stomas formed in the mixed group of 25 patients who received chemoradiotherapy without boost. One patient required a defunctioning colostomy prior to chemoradiotherapy because of severe anal pain and faecal incontinence. One patient developed a rectovaginal fistula during chemoradiotherapy causing treatment to be interrupted. One patient developed a sigmoid stricture after chemoradiotherapy requiring a Hartmann’s procedure. Two patients received an abdominoperineal resection of rectum for local recurrence (one with additional vulvectomy). None of these patients wished to have a defunctioning stoma because of poor anal function. All patients were asked about anal function in the follow-up clinic, and all were satisfied with their anal function. No formal score such as the Wexner incontinence score was used [6].

## Discussion

The main aims in the treatment of SCC of the anus are to achieve a high rate of cure without a permanent stoma and adequate (from the patients perspective) anal function [7]. We have shown that this is possible to achieve in an unselected population, but over 20 % of our patients seen from this defined population were not suitable for radical chemotherapy. In order to achieve these goals, it is important that an initial defunctioning colostomy is not performed unless there is a fistula (in particular a recto-vaginal fistula) or distressing anal pain. In addition, a high enough dose of radiotherapy is needed for advanced tumours (large T2, T3 and T4) to obtain a high rate of local control [8, 9]. A brachytherapy boost [10, 11] to a hemicircumference of the anal canal achieves the higher dose without giving the full dose to the hemicircumference not implanted. This is impossible to achieve by any other method including intensity modulated radiotherapy [12–16] which is not cost-effective [17]. A major disadvantage of anal brachytherapy in general radiotherapy practise is that relatively few radiation oncologists are trained in this technique.

In this study, the number of patients treated was small compared to the six published randomised controlled trials [2, 3, 5, 9, 18, 19]. However, there were only three local recurrences after radical chemoradiotherapy with a brachytherapy or electron boost in 52 patients, showing that a high rate of local control can be obtained in patients resident in a defined population.

Close collaboration between specialist colorectal cancer surgeons with an interest in anal cancer and oncologists with a specialist interest in anal cancer at multidisciplinary team meetings can result in the smallest achievable percentage of stomas created prior to treatment. It is important that in addition to reporting permanent colostomy rates, cancer-related and treatment-related colostomy rates are also reported [20–24]. 

A low rate of stoma formation occurred in this study especially in the brachytherapy group. Only 2 of 52 patients had a stoma formed prior to treatment because of anal pain or fistula, and these stomas were not reversed. The three patients with local recurrence all had salvage abdomino-perineal excision of rectum performed, and all three had further recurrence. In the UNICANCER ACCORD 3 trial [9], patients treated initially with definitive colostomy were not eligible for the study but a temporary colostomy was allowed for severe symptoms or fistula if a reversal of the colostomy was reasonably expected. The 5-year colostomy-free survival was 73.7–77.8 % in this trial. All patients in this trial received a boost either with ^192^Ir brachytherapy or electron beam therapy. No information was given concerning reversal of the temporary colostomies. The colostomy-free survival in this trial was higher than expected in the standard dose boost with no induction chemotherapy. The patients were recruited between 1999 and 2005. In our retrospective study, the patients were treated between 2000 and 2011. It is possible that in our single-centre study, it was easier to limit the use of a stoma than in a multicentre randomised trial, and consequently, the cancer-related stoma rate at 5 years was 3 % for the brachytherapy group and 20 % for the electron group with no treatment-related stomas at 5 years.

The assessment of anal function however during the follow-up of patients is difficult for several reasons:

1. The estimated prevalence of anal incontinence (including double incontinence) in the normal population (excluding people in care homes) over the age of 65 years is 5–10 % in men and 10–15 % in women [6, 25].
2. The major cause of damage to the anal canal and sphincter is probably caused by the anal cancer and may wholly or partly be permanent.
3. Unless there is considerable fibrosis after treatment, it may be difficult to attribute poor anal function to the effect of treatment.
4. Patients find discussing anal incontinence embarrassing and may play-down their symptoms. In this study, all the patients without a stoma or local recurrence were satisfied with their anal function.

## Conclusions

A low rate of stoma formation can be achieved in the treatment of anal cancer with chemoradiotherapy if the number of stomas prior to treatment is kept to a minimum and the treatment protocol used leads to a low rate of local recurrence. The indication in our series for a stoma prior to chemoradiotherapy was either severe anal pain with or without faecal incontinence or a rectovaginal fistula. In all other patients, a stoma prior to chemoradiotherapy was avoided.
